# Health care utilisation amongst older adults with sensory and cognitive impairments in Europe

**DOI:** 10.1186/s13561-017-0183-1

**Published:** 2017-12-01

**Authors:** David G. Lugo-Palacios, Brenda Gannon

**Affiliations:** 1 Centre for Health Economics, University of Manchester, 4.306 Jean McFarlane Building, Oxford Road, M13 9PL, Manchester, UK; 2 Centre for the Business and Economics of Health, The University of Queensland, Faculty of Business, Economics and Law, QLD, St Lucia, 4072 Australia

**Keywords:** Ageing, cognitive impairment, Sensory impairment, Health care utilisation, Correlated random effects, SHARE, I110, I120, I140

## Abstract

Worldwide, the high prevalence of multiple chronic conditions amongst older population has led to increased utilisation of health care and rising associated costs, becoming a major public health concern. Hearing, vision and cognitive disorders are common chronic conditions amongst older Europeans and recent studies have documented its high co-occurrence. While it has been shown separately that suffering either mental disorders or sensory (hearing and vision) impairments is associated with higher health care utilisation, the association between health care utilisation and the interaction of these conditions has received little attention in the literature. Therefore, using four waves of the Survey of Health, Ageing and Retirement in Europe (SHARE), this study applies the correlated random effects method to the negative binomial and finite mixture models to analyse the extent to which the interaction of cognitive and sensory impairments is associated with health care use. We found that individuals with cognitive impairment tend to have more hospitalisations. The finite mixture approach indicates a positive association between sensory impairment and the number of hospitalisations amongst low users of health care. Additionally, our findings suggest a positive association between suffering both impairments at the same time and the number of doctor and GP visits.

## Background

The high prevalence of multiple chronic conditions amongst older population has led to decreased quality of life, to increased utilisation of health care services and rising associated costs across the world; thus, becoming a major public health concern [1, 2]. Even in the coexistence of chronic conditions, individual diseases dominate health care delivery. However, the use of many services to manage individual diseases can become duplicative, inefficient and, in some cases, unsafe for patients [3]. Hearing, vision and cognitive disorders are common chronic conditions amongst older Europeans and recent studies have documented its high co-occurrence [4–6]. Moreover, significant statistical associations between sensory impairment and cognitive decline have been found in previous research and several hypothesis have been proposed to account for the relationship between them [7, 8]. While it has been shown separately that suffering either mental disorders or sensory impairments is associated with higher utilisation of health care resources, the association between health care utilisation and the interaction of these conditions has received little attention in the literature [9–11]. Therefore, in an effort to better understand health care utilisation amongst older population suffering sensory and cognitive impairments, this study examines the extent to which older Europeans with both impairments visit any medical doctor, a general practitioner (GP) and a hospital in comparison with individuals with only sensory impairment, with only cognitive impairment or with none of these conditions.

Grossman considers that an individual, seen both as consumer and producer of its own health, inherit an initial stock of health that depreciates with age and can be increased by investment [12, 13]. Time and other inputs, such as health care and regular physical activity, are used to produce healthy time. Hence, the net investment in the stock of health equals gross investment minus depreciation. In the original Grossman model, while the rates of depreciation are exogenous, they depend on age. As the depreciation rate increases, the marginal cost of healthy days increases as well and, thus, a utility-maximiser individual will choose a lower stock of health. An increase in the shadow price of capital, caused by a higher depreciation rate, reduces not only the demand for health capital, but also the amount of health capital supply to the individual by a given amount of gross investment [14, 15]. Nevertheless, assuming that the demand for health is inelastic, the demand for health inputs (i.e. health care) will increase with age to compensate part of the reduction in health capital. The negative relationship between the demand for health and the one for health care predicts that people who are older and less healthy will increase their consumption of health care [14].

Previous studies have shown that hearing, vision and cognitive impairments amongst older adults impact negatively on functional independence, mental health and reduce quality of life, increasing the need for support services [11, 16]. Furthermore, it has been postulated that sensory impairment and cognitive decline may both be the result of age-related changes in a shared factor, such as degeneration of central nervous structures [7]. In addition, Kumagai and Ogura (2014) found that increasing the intensity of regular physical activity has positive effects on health stock suggesting that health enhancement behaviours may reduce the need and use of health care [17]. Therefore, holding the rest constant*,* it is expected that older population with sensory and cognitive impairments with no regular physical activity (higher depreciation rate and lower stock of health capital) use more health services than older population with only one or none of these impairments.^1^ The objective of this paper is to test the validity of this hypothesis amongst older Europeans.

### Data

The analysis uses data from waves 1, 2, 4 and 5 of the Survey of Health, Ageing and Retirement in Europe (SHARE), Release 5.0.0 collected between 2004 and 2013 [18]. SHARE is a multidisciplinary cross-national panel survey representative at the national level that collects data on health, socio-economic status and family networks of more than 123,000 non-institutionalised individuals aged 50 or over (and their spouses) from 20 European countries. Data from the third wave are excluded in this analysis since it only collects the retrospective histories of the respondents and does not contain data on recent health conditions and health care utilisation. The present study only uses data from nine countries present in all the available waves (Austria, Belgium, Denmark, France, Germany, Italy, the Netherlands, Spain, and Switzerland).

Health care utilisation within the last 12 months is examined separately with three different variables: number of times the respondent has seen a medical doctor, the number of these contacts that were visits to a GP, and the number of times that the respondent was a patient in a hospital overnight. Unfortunately, in wave 5 the number of times that a person visited the GP was not separated from the overall visits to a medical doctor. For this reason, wave 5 was excluded from the analysis that takes visits to the GP as the dependent variable.

This paper uses measures of episodic memory and verbal fluency as indicators of cognitive function [19]. The episodic memory test in SHARE consists of a verbal recall of a list of ten words (in waves 1 and 2 the same list was used whereas in waves 4 and 5 respondents were assigned randomly to one of four sets of ten words). This test is implemented two times: immediately after the respondent hears the complete list (immediate recall) and at the end of the cognitive function module (delayed recall).

The number of words remembered correctly from both the immediate and the delayed recall are added generating a new episodic memory variable that ranges between 0 and 20 [20]. In the literature on cognitive decline, a memory score of 1.5 standard deviations below the age-specific mean has been considered an indicator of mild cognitive impairment [21, 22]. Therefore, this method is applied within each country using the statistics of the episodic memory variable to generate a binary variable that identifies individuals with mild cognitive impairment. Due to a very low cut-off point for individuals aged 75 or older (due to a relatively low number of observations), a different cut-off was defined for these individuals. For this age group the mean and standard deviation taken into consideration are not age specific and the age group (75 and older) statistics are used instead. Moreover, instead of taking a 1.5 standard deviation below the average as the cut-off point to indicate mild cognitive impairment, only one standard deviation from the age group mean is considered.

In the verbal fluency test, respondents were asked to name as many animals as possible in 60 s. Since naming less than 15 different animals (excluding repetitions) is suggestive of dementia and naming 15 or more are considered normal, a variable indicating if the respondent has a verbal fluency problem was created if respondents failed to name at least 15 correct words [23].

A third dummy variable classifies an individual as cognitive impaired if he/she has either an episodic memory problem or a verbal fluency problem, as defined above.

Sensory impairment is measured using self-reported vision and hearing quality. In the case of vision quality, two self-reported measures were used to identify impairment: distance eyesight and reading eyesight (using glasses or contact lenses as usual). Individuals who responded their eyesight was fair or poor (as opposed to excellent, very good or good) are categorised as visually impaired [24]. In the same line, participants that answered that their hearing was fair or poor (using hearing aid as usual) are classified as having a hearing impairment. Individuals with either vision or hearing impairment are then considered to be sensory impaired.

Along with the sensory and cognitive impairment indicator variables, the interaction term between these two variables are the main covariates of interest in this study as they will allow the investigation of the extent to which individuals with these impairments are using health care services. The choice of additional regressors follows the Andersen model that considers that health care utilisation is a function of an individual’s predisposing, enabling and need characteristics, as well as of his/her health behaviours [25]. The predisposing characteristics considered are age and age squared treated as continuous variables, a dummy variable indicating if the individual is married, as well as the person’s sex, immigration status and education level. The latter was categorised into three levels (none/primary, secondary, and tertiary) using the International Standard Classification of Education codes reported in SHARE [26]. Household income in its logarithmic form, adjusted for purchase power, and employment status (employed or not employed) are used as enabling characteristics.^2^ Apart from the variables indicating sensory and cognitive impairments, the need factors included are the number of chronic diseases reported and the Activity Daily Living (ADL) scale that measures the individual functional status. The ADL scale is treated as a continuous variable ranging between 0 (no impediment) and 6 (total dependence). To account for health behaviours, both risky and health enhancement activities were considered in this study: two variables are introduced to indicate whether the individual smokes at the moment of the interview and whether the individual drinks alcohol more than three days a week; it was considered that an individual undertook health enhancement activities if he/she engaged more than once a week in vigorous physical activity. Finally, SHARE wave dummy variables are also considered.

The observations with missing values for at least one of the variables mentioned are dropped. Likewise, as described below, only individuals with two or more observations in the panel are included in the analysis. The resulting data set is an unbalanced panel with 85,473 observations from 32,229 individuals. Table 1 shows the descriptive statistics of the data set analysed. SHARE respondents had on average 7 visits to the doctor, 4.7 visits to the GP and 0.24 hospitalisations during the whole period of study. Pooling the four SHARE waves, 28% interviews identified individuals with cognitive impairment, 38% with sensory impairment, and 14% with both impairments at the same time. Furthermore, 57.9% of the 32,229 individuals were considered sensory impaired at least once in the study period; the percentages for individuals with at least one report of being cognitively impaired and at least one report of having both impairments at the same time are 42.7% and 24.2%, respectively (not shown).

**Table 1:** Descriptive statistics

Variable	Observations	Mean	Std. Dev.	Min	Max
*Health care utilisation*					
Doctor visits	85,147	7.00	9.59	0	98
GP visits	59,965	4.73	7.01	0	98
Hospitalisations	85,473	0.24	0.80	0	10
*Predisposing factors*					
Age	85,473	65.69	9.73	50	104
Male	85,473	0.45	0.50	0	1
Married	85,473	0.72	0.45	0	1
Immigrant	85,473	0.08	0.27	0	1
Primary education	85,473	0.27	0.44	0	1
Secondary education	85,473	0.51	0.50	0	1
Tertiary education	85,473	0.22	0.41	0	1
*Enabling factors*					
Employed	85,473	0.26	0.44	0	1
ln(income_ppp)	85,473	10.03	1.40	−5.62	15.99
*Health behaviours*					
Present smoker	85,473	0.18	0.38	0	1
Regular alcohol consumption	85,473	0.37	0.48	0	1
Regular physical activity	85,473	0.34	0.47	0	1
*Need factors*					
ADL scale	85,473	0.19	0.72	0	6
No. of chronic diseases	85,473	1.40	1.31	0	11
Cognitive impairment	85,473	0.28	0.45	0	1
Sensory impairment	85,473	0.38	0.48	0	1
Sense_Cog impairment	85,473	0.14	0.34	0	1
*Country dummies*					
Austria	85,473	0.12	0.32	0	1
Germany	85,473	0.07	0.26	0	1
Netherlands	85,473	0.10	0.30	0	1
Spain	85,473	0.11	0.31	0	1
Italy	85,473	0.11	0.32	0	1
France	85,473	0.15	0.36	0	1
Switzerland	85,473	0.09	0.28	0	1
Belgium	85,473	0.16	0.37	0	1
Denmark	85,473	0.09	0.28	0	1
*Wave dummies*					
SHARE Wave 1	85,473	0.18	0.38	0	1
SHARE Wave 2	85,473	0.21	0.41	0	1
SHARE Wave 4	85,473	0.31	0.46	0	1
SHARE Wave 5	85,473	0.30	0.46	0	1
Number of waves	85,473	2.91	0.87	2	4
*Number of zero counts*					
	Observations	Zeros	Percentage of zeros		
Doctor visits	85,147	8784	10.32%		
GP visits	59,965	9485	15.82%		
Hospitalisations	85,473	72,796	85.17%		

Table 1 also displays the zero counts in the variables measuring the use of health care. The percentage of zeros is 10.3% for doctor visits, 15.8% for GP visits, and 85.2% for hospitalisations.

## Methods

This paper analyses health care utilisation amongst SHARE population over time. A key decision when analysing panel data is to choose how to handle the panel heterogeneity caused by the time-invariant individual-specific components. One option is to treat individual heterogeneity as an unobserved random variable uncorrelated with the regressors (random effects, RE). The problem with this approach is that if the no-correlation assumption between the individual effects and the covariates is violated, then estimates obtained from a RE model will not be consistent. A popular alternative in the applied micro-econometrics literature is the use of fixed effects (FE) models as they allow the individual-specific effects and the regressors to be correlated [27]. In a FE model, individual effects are eliminated by either first differencing or applying the deviations-from-mean transformation, but time-invariant regressors are also swept out with this transformation [28]. This can be problematic if time-invariant covariates or with little variance across the panel are of special interest. There is, however, an alternative to FE models that can yield consistent estimates while allowing the inclusion of time-invariant regressors: conditionally correlated random effects (CCRE) [29, 30]. This approach was originally proposed for linear regression, but has been adapted for count data models [31].

This paper follows the CCRE approach by estimating two different models. The first is a conditionally correlated random effects negative binomial (CCRENB) model and the second is a latent class negative binomial model for panel data with correlated random effects (LCNB_CRE).

### The CCRENB model

Table 1 suggests that the three health care variables used in this paper are overdispersed (conditional variance significantly higher than the conditional mean). Negative binomial models are often used in this case as they can accommodate overdispersed data and may improve efficiency in estimation compared to Poisson models [27].

The negative binomial random effects (RE) model was first proposed in [32] by supposing that *y*_*it*_ is independent and identically distributed negative binomial with quadratic variance function with parameters *α*_*i*_ *λ*_*it*_ and *ϕ*_*i*_, where *α*_*i*_ is a time-invariant individual-specific random effect, ${\lambda}_{it}=\exp \left({x}_{it}^{\prime}\beta \right)$, *ϕ*_*i*_ is a dispersion factor, *x*_*it*_ are the exogenous covariates, and *β* is the vector of regression parameters. This implies that *y*_*it*_ has mean *α*_*i*_ *λ*_*it*_/*ϕ*_*i*_ and variance (*α*_*i*_ *λ*_*it*_/*ϕ*_*i*_) × (1 + *α*_*i*_/*ϕ*_*i*_) [27]. If (1 + *α*_*i*_/*ϕ*_*i*_)-1 is a beta-distributed random variable with parameters (a, b), then the joint density of individual *i* ‘s health care utilisation is

$$ f\left({y}_{it}|{X}_{it},\beta, a,b\right)=\prod \limits_{t=1}^T\left(\frac{\varGamma \left({\lambda}_{it}+{y}_{it}\right)!}{\varGamma \left({\lambda}_{it}\right)!\varGamma \left({y}_{it}+1\right)!}\right)\times \frac{\varGamma \left(a+b\right)\varGamma \left(a+\sum \limits_t{\lambda}_{it}\right)\varGamma \left(b+\sum \limits_t{y}_{it}\right)}{\varGamma (a)\varGamma (b)\varGamma \left(a+b+\sum \limits_t{\lambda}_{it}+\sum \limits_t{y}_{it}\right)}\kern2.75em (1) $$

where *y*_*it*_ is the count of doctor visits (or hospitalisations) and *Γ*(.) is the gamma function. Eq. (1) is the basis for maximum likelihood estimation of *β*, a and b. As any other RE model, the estimated coefficients are only consistent if the RE are uncorrelated with the covariates. The no-correlation assumption between the individual effects and the covariates can be relaxed in a RE framework by applying the Mundlak-Chamberlain method to the count data case. This is done by allowing the time-invariant individual effect to depend on the average of individual effects**:**

2 $$ {\alpha}_i=\exp \left(\overline{x}{\prime}_i\varphi +{\varepsilon}_i\right) $$

where ${\overline{x}}_i$ is the time-average of the covariates and *ε*_*i*_ may be interpreted as unobserved heterogeneity that is uncorrelated with the regressors [31]. The conditional mean of *y*_*it*_ can then be expressed by

3 $$ E\left[\left({y}_{it}|{X}_{it},{\alpha}_i\right)=\exp \left({x}_{it}^{\prime}\beta +\overline{x}{\prime}_i\varphi +{\varepsilon}_i\right)\right] $$

Equation (3) can be estimated as an RE model, with $\overline{x}{\prime}_i$ as an additional regressor [31]. Only individuals observed at least in two different waves are included in the analysis as changes over time are unobserved for individuals with only one observation [33].

Although the negative binomial model is superior to the Poisson in that it allows for overdispersion, its use might be inadequate in analysing data with excess zeros [34]. While on this paper excess zeros is not a concern in the case of doctor and GP visits, it is in the case of hospitalisations. Therefore, in addition to the CCRENB model, this paper also considers a methodology that takes this excess of zeros into account.

### The LCNB_CRE model

An alternative approach to handle heterogeneity in a count data framework is to use latent class (or finite mixture) models. These models can provide an effective way of handling both excess zeros and overdispersion in count models [31, 35, 36]. This method assumes that the sample of individuals is drawn from a population divided in C different latent classes with a probability *π*_*j*_ of belonging to the jth latent class, where ${\sum}_{j=1}^C{\pi}_{ij}=1,\kern0.5em 0\le {\pi}_{ij}\le 1,\kern0.5em j=1,\dots, C.$ It is also assumed that the variable of interest (*y*_*it*_) follows a different underlying distribution within each latent class. Here, the latent classes are assumed to be based on the individual’s latent long-term health status which may not be well captured by proxy variables. In this sense, the latent class framework represents unobserved time-invariant heterogeneity [37].

Following Jones et al. (2013)**,** the panel structure is accounted for in the formulation of the mixture of probabilities and densities [38]. Let *y*_*it*_ represent the number of doctor visits (or hospitalisations) in year t. Conditional on the class that individual *i* belongs to, *y*_*it*_ has density *f*_*j*_(*y*_*it*_| *x*_*it*_; *θ*_*j*_) with *θ*_*j*_ vectors of parameters specific to each class. Given class j, the joint density of *y*_*it*_ over the observed periods (*T*_*i*_) is a product of *T*_*i*_ independent densities *f*_*j*_(*y*_*it*_| *x*_*it*_; *θ*_*j*_). Unconditionally on the latent class, the joint density of ${y}_i=\left[{y}_{i1},\dots, {y}_{i{T}_i}\right]$ is given by

$$ g\left({y}_i|{x}_i;{\pi}_{i1},\dots, {\pi}_{iC;}{\theta}_1,\dots, {\theta}_C\right)=\sum \limits_{j=1}^C{\pi}_{ij}\prod \limits_{t=1}^{T_i}{f}_j\left({y}_{it}|{x}_{it};{\theta}_j\right)(4) $$

Class membership probabilities are commonly taken in the literature as fixed parameters to be estimated along with *θ*_*j*_ [37]. This is analogous to a RE specification that assumes no correlation between individual heterogeneity and the regressors [38]. To relax this assumption, the Mundlak-Chamberlain approach is followed again, but now modelling class membership as a function of time-invariant individual characteristics using a multinomial logit [37]:

$$ {\pi}_{ij}=\frac{\mathit{\exp}\left(\overline{x_i}{\gamma}_j\right)}{\sum_{g=1}^C\mathit{\exp}\left(\overline{x_i}{\gamma}_g\right)},\kern0.5em j=1,\dots, C\kern7.75em (5) $$

with *γ*_*C*_ = 0, $\overline{x_i}$ defined as in eq. (2), and defining each of the j density functions in [4] in the same way as eq. (1). The vectors of parameters *θ*_*j*_ and *γ*_*j*_are estimated jointly by maximum likelihood using the Broyden-Fletcher-Goldfarb-Shanno quasi-Newton algorithm. Following most applications of the latent class approach in health care, this paper defines C = 2 and based on the predicted number of counts these classes are referred as “low users” and “high users” of health care. STATA 14 is used in all estimations [39].

## Results

### Regression results of the CCRENB model

Results of the CCRENB model are presented in the form of incidence-rate-ratios (IRR) in Table 2. IRRs significantly lower than one for a given covariate are interpreted as indicative of a negative association between the covariate in question and the type of care analysed; IRRs significantly higher than one suggest a positive relationship. Results in this paper are reported as IRRs instead of marginal effects, as estimation of the latter would require assumptions that would undermine the existence of heterogeneity across individuals; specifically, marginal effects would be estimated by assuming that all the random effects are zero [40].

**Table 2:** Health care utilisation in SHARE: negative binomial conditionally correlated random effects model

	Doctor Visits		GP visits		Hospitalisations	
	[1]	[2]	[3]	[4]	[5]	[6]
*Predisposing factors*						
Age	0.970***	0.969***	0.951***	0.950***	0.987	0.986
	[0.009]	[0.009]	[0.011]	[0.011]	[0.033]	[0.033]
Age squared	1.000	1.000	1.000	1.000	1.000	1.000
	[0.000]	[0.000]	[0.000]	[0.000]	[0.000]	[0.000]
Male	0.928***	0.927***	0.963***	0.963***	1.198***	1.197***
	[0.008]	[0.008]	[0.009]	[0.009]	[0.026]	[0.026]
Married	1.009	1.008	1.006	1.005	1.089	1.089
	[0.024]	[0.024]	[0.033]	[0.033]	[0.092]	[0.092]
Immigrant	1.045***	1.045***	1.029*	1.028	1.021	1.020
	[0.015]	[0.015]	[0.017]	[0.017]	[0.038]	[0.038]
Secondary education	0.993	0.992	0.956***	0.955***	1.035	1.032
	[0.010]	[0.010]	[0.012]	[0.012]	[0.028]	[0.028]
Tertiary education	1.043***	1.043***	0.903***	0.903***	1.022	1.022
	[0.013]	[0.013]	[0.014]	[0.014]	[0.036]	[0.036]
*Enabling factors*						
Employed	0.904***	0.904***	0.936***	0.936***	0.865***	0.864***
	[0.013]	[0.013]	[0.018]	[0.018]	[0.048]	[0.048]
ln(Income_ppp)	1.014***	1.014***	1.011***	1.011***	0.999	0.999
	[0.003]	[0.003]	[0.003]	[0.003]	[0.010]	[0.010]
*Health behaviours*						
Present smoker	0.877***	0.877***	0.914***	0.914***	0.587***	0.587***
	[0.015]	[0.015]	[0.020]	[0.020]	[0.034]	[0.034]
Regular alcohol consumption	0.956***	0.956***	0.952***	0.952***	0.844***	0.844***
	[0.010]	[0.010]	[0.012]	[0.012]	[0.031]	[0.031]
Regular physical activity	0.937***	0.937***	0.951***	0.951***	0.832***	0.832***
	[0.008]	[0.008]	[0.011]	[0.011]	[0.026]	[0.026]
*Need factors*						
ADL scale	1.033***	1.033***	1.032***	1.032***	1.156***	1.156***
	[0.005]	[0.005]	[0.006]	[0.006]	[0.017]	[0.017]
Number of chronic diseases	1.117***	1.117***	1.093***	1.093***	1.185***	1.185***
	[0.004]	[0.004]	[0.005]	[0.005]	[0.013]	[0.013]
Cognitive impairment	0.991	0.981*	1.001	0.98	1.081**	1.075*
	[0.009]	[0.011]	[0.012]	[0.014]	[0.035]	[0.044]
Sensory impairment	1.017**	1.009	1.007	0.989	1.047*	1.042
	[0.008]	[0.009]	[0.010]	[0.012]	[0.029]	[0.034]
Sense_cog impairment		1.023		1.049**		1.014
		[0.015]		[0.020]		[0.053]
Austria	1.311***	1.310***	1.146***	1.145***	1.877***	1.876***
	[0.024]	[0.024]	[0.025]	[0.025]	[0.088]	[0.088]
Germany	1.567***	1.566***	1.263***	1.262***	1.419***	1.417***
	[0.031]	[0.031]	[0.029]	[0.029]	[0.074]	[0.074]
Netherlands	1.085***	1.084***	0.905***	0.904***	0.921	0.92
	[0.020]	[0.020]	[0.020]	[0.020]	[0.049]	[0.049]
Spain	1.252***	1.252***	1.132***	1.132***	0.755***	0.757***
	[0.024]	[0.024]	[0.026]	[0.026]	[0.041]	[0.041]
Italy	1.375***	1.375***	1.314***	1.314***	0.836***	0.837***
	[0.026]	[0.026]	[0.029]	[0.029]	[0.044]	[0.044]
France	1.540***	1.539***	1.467***	1.465***	1.178***	1.176***
	[0.026]	[0.026]	[0.030]	[0.030]	[0.056]	[0.056]
Switzerland	1.131***	1.131***	0.966	0.966	1.350***	1.349***
	[0.022]	[0.022]	[0.023]	[0.023]	[0.072]	[0.072]
Belgium	1.564***	1.563***	1.497***	1.495***	1.175***	1.173***
	[0.026]	[0.026]	[0.030]	[0.030]	[0.054]	[0.054]
SHARE Wave 2	1.109***	1.109***	1.108***	1.108***	1.141*	1.142*
	[0.023]	[0.023]	[0.027]	[0.027]	[0.085]	[0.085]
SHARE Wave 4	1.243***	1.245***	1.311***	1.315***	1.139	1.140
	[0.069]	[0.069]	[0.089]	[0.089]	[0.227]	[0.228]
SHARE Wave 5	1.406***	1.408***	.	.	1.227	1.229
	[0.101]	[0.101]	.	.	[0.316]	[0.317]
N	85,147	85,147	59,965	59,965	85,473	85,473
ll	−239,098.39	−239,094.79	−146,884.11	−146,879.70	−45,976.20	−45,974.52
AIC	478,284.78	478,281.57	293,854.22	293,849.40	92,040.40	92,041.05
BIC	478,696.28	478,711.77	294,241.29	294,254.47	92,452.06	92,471.42

*IRR* Incidence-rate ratios. * *p* < 0.10, ** *p* < 0.05, *** *p* < 0.01. IRR of time-averaged covariates are not shown. Standard errors in brackets

Two separate regressions are carried out for each type of health care: one including the interaction term of sensitive and cognitive impairments (even columns) and other without this interaction (odd columns). Bayesian Information Criteria (BIC) is reported as measures of goodness of fit, models with smaller values of BIC are preferred.

The models that include the Sense-Cog interaction show that having a cognitive but no sensory impairment is associated with 1.9% fewer doctor visits and 7.5% more hospitalisations at the 10% significance level. Column [4] indicates that individuals with both cognitive and sensory impairments tend to have 4.9% more GP visits than individuals with none of these impairments at the 5% significance level.

When the Sense-Cog interaction is not included in the model, the estimates of cognitive and sensory impairment are interpreted considering the rest of the other factors constant. Hence, ceteris paribus, having a cognitive impairment is not significantly associated with doctor and GP visits, but individuals with this impairment tend to have 8.1% more hospitalisations than individuals without this impairment. Columns [1] and [5] show that sensory impairment is significantly associated at the 5% level with 1.7% more doctor visits and at the 10% significance level with 4.7% more hospitalisations.

The associations found for the rest of the covariates are robust to the inclusion of the Sense-Cog interaction term. The number of chronic diseases is strongly associated with the use of health care; suffering an additional chronic disease is associated with 12% more doctor visits, 9% more GP visits and 19% more hospitalisations. The ADL scale is also positively associated with health care utilisation. Unhealthy behaviours are negatively associated with the use of health care: present smokers tend to have 12% fewer visits to the doctor and 41% fewer hospitalisations. A health enhancement behaviour is also negatively associated with the use of health care: being physically active more than one day a week is associated with 6% fewer doctor visits, 5% fewer GP visits and nearly 17% fewer hospitalisations. The direction and the magnitude of the association of health care utilisation with other factors depend on the type of care analysed. For example, males tend to have fewer visits to the doctor and to the GP, but more hospitalisations; and higher income level is only positively associated with more visits to the doctor and to the GP, but it is not significantly associated with the number of hospitalisations.

For presentation purposes the IRRs of the average across the panel of the time-varying covariates were not reported in Table 2, but they are available in the Appendix. The estimated coefficients can be interpreted as long-term associations with the use of health care. In this sense, the long-term association of being cognitively impaired is positive and significantly associated with doctor and GP visits. In the same line, smoking is positively associated in the long term with hospitalisations. The strongest long-term association displayed in Table 5 in Appendix is the one between the number of chronic conditions and GP visits.

### Regression results of the LCNB_CRE model

Table 3 shows the estimation results of the LCNB_CRE model. By design, these models are estimated using balanced panels [38]. Consequently, a significant amount of observations are lost with the implementation of this method. Given that the BIC in the CCRENB model favours the specification without the Sense-Cog interaction, only this is considered in Table 3 but the unrestricted alternative is reported in the Appendix.

**Table 3:** Health Care utilisation in SHARE: latent class negative binomial conditionally correlated random effects model

	Doctor Visits		GP visits		Hospitalisations	
	[1]	[2]	[3]	[4]	[5]	[6]
	*Low users*	*High users*	*Low users*	*High users*	*Low users*	*High users*
*Predisposing factors*						
Age	1.012	1.021	1.016	0.989	1.055	1.000
	[0.013]	[0.014]	[0.015]	[0.013]	[0.048]	[0.056]
Age squared	1.000	1.000	1.000	1.000	1.000	1.000
	[0.000]	[0.000]	[0.000]	[0.000]	[0.000]	[0.000]
Male	0.909***	0.99	0.967*	1.013	1.239***	1.186**
	[0.016]	[0.019]	[0.019]	[0.020]	[0.069]	[0.089]
Married	1.057**	1.000	1.032	0.983	1.045	1.155
	[0.025]	[0.023]	[0.027]	[0.023]	[0.081]	[0.132]
Immigrant	1.042	0.978	0.977	0.996	1.212*	0.738*
	[0.037]	[0.038]	[0.035]	[0.037]	[0.123]	[0.125]
Secondary education	1.029	1.008	0.989	0.917***	1.048	1.062
	[0.023]	[0.023]	[0.025]	[0.021]	[0.072]	[0.097]
Tertiary education	1.077***	1.082**	0.921***	0.857***	0.964	1.154
	[0.029]	[0.034]	[0.028]	[0.028]	[0.082]	[0.137]
*Enabling factors*						
Employed	0.922***	0.859***	0.951*	0.845***	0.872	0.818
	[0.024]	[0.025]	[0.026]	[0.027]	[0.103]	[0.105]
ln(income_ppp)	1.025***	0.995	1.014*	0.993	1.001	0.98
	[0.008]	[0.006]	[0.008]	[0.006]	[0.024]	[0.025]
*Health behaviours*						
Present smoker	0.833***	0.938**	0.835***	0.966	0.911	0.744***
	[0.023]	[0.024]	[0.024]	[0.026]	[0.098]	[0.082]
Regular alcohol consumption	0.964**	0.876***	0.962**	0.913***	0.858**	0.837**
	[0.018]	[0.017]	[0.019]	[0.019]	[0.053]	[0.074]
Regular physical activity	0.883***	0.920***	0.907***	0.908***	0.842***	0.761***
	[0.015]	[0.018]	[0.017]	[0.019]	[0.054]	[0.065]
*Need factors*						
ADL scale	1.042**	1.129***	1.012	1.101***	1.272***	1.120**
	[0.017]	[0.015]	[0.023]	[0.012]	[0.040]	[0.060]
Number of chronic diseases	1.339***	1.144***	1.338***	1.116***	1.329***	1.090***
	[0.010]	[0.008]	[0.012]	[0.008]	[0.028]	[0.034]
Cognitive impairment	0.942***	1.019	0.978	1.063***	0.890*	1.164*
	[0.020]	[0.021]	[0.024]	[0.022]	[0.062]	[0.102]
Sensory impairment	0.989	0.992	0.988	1.01	1.167***	0.946
	[0.017]	[0.017]	[0.019]	[0.019]	[0.066]	[0.072]
*Country dummies*						
Austria	1.345***	1.224***	1.112**	1.338***	2.069***	1.073
	[0.069]	[0.059]	[0.055]	[0.068]	[0.260]	[0.196]
Germany	1.984***	1.652***	1.346***	1.496***	1.924***	0.908
	[0.071]	[0.080]	[0.054]	[0.074]	[0.234]	[0.159]
Netherlands	1.133***	1.135***	0.948	0.867***	0.894	0.781
	[0.040]	[0.051]	[0.040]	[0.042]	[0.115]	[0.121]
Spain	1.419***	1.158***	1.182***	1.337***	0.704**	0.616***
	[0.057]	[0.053]	[0.058]	[0.064]	[0.097]	[0.102]
Italy	1.529***	1.491***	1.156***	1.853***	0.837	0.629***
	[0.060]	[0.065]	[0.050]	[0.086]	[0.109]	[0.100]
France	1.673***	1.271***	1.610***	1.333***	1.276**	0.954
	[0.057]	[0.057]	[0.060]	[0.062]	[0.151]	[0.143]
Switzerland	1.124***	1.151**	0.921*	1.100	1.458***	1.396
	[0.049]	[0.068]	[0.045]	[0.070]	[0.198]	[0.290]
Belgium	1.649***	1.426***	1.504***	1.569***	1.291**	0.734**
	[0.052]	[0.057]	[0.053]	[0.067]	[0.014]	[0.014]
N	28,328		26,571		28,736	
ll	−79,603.25		−65,226.65		−14,502.19	
AIC	159,334.50		130,581.31		29,132.39	
BIC	159,773.88		131,035.00		29,572.68	

*IRR* Incidence-rate ratios. * *p* < 0.10, ** *p* < 0.05, *** *p* < 0.01. IRR of time-averaged covariates are not shown. Standard errors in brackets

Having a cognitive impairment is associated with 6% fewer doctor visits amongst low users, but has no significant association with high users. Contrary to this finding, but in the same direction to what was found in the CCRENB model, this impairment is associated with more hospitalisations amongst high users; however, amongst low users this association is negative. The insignificant association between GP visits and being cognitively impaired found in the CCRENB still holds amongst low users, but not for frequent users of GP services as having this impairment is associated with 6% more visits.

Column [5] shows that there is strong evidence supporting that sensory impairment is associated with 17% more hospitalisations amongst infrequent users of hospital services. The positive association amongst individuals with this impairment and visits to the doctor found in Table 2 is not supported anymore in the LCNB_CRE model.

With the only exception of the ADL scale amongst infrequent users of GP services, the strong, positive and significant association between health care utilisation and the rest of the need factors found in the CCRENB is still present in the latent class approach. Also consistent with the findings of the previous model, being employed and both unhealthy and health enhancement behaviours are negatively associated with all types of health care utilisation. In the CCRENB model income was positively associated with doctor and GP visits, now this association is only present amongst low users of health care.

Interestingly, the association found between immigration status and the number of hospitalisations depends on whether the individual is a frequent or an infrequent user. Being an immigrant is associated with 21% more hospitalisations amongst lower users but with 26% fewer hospitalisations amongst high users.

Table 4 reports the sample average of predicted probability of belonging to the high user class for each type of health care as well as the expected number of visits for each class and type of care.

**Table 4:** Predicted probability and expected number of visits by class

	Prob. High user	E[visits] if low user	E[visits] if high user
Doctor Visits	0.43	4.07	10.54
		[2.77]	[4.19]
GP visits	0.45	2.62	7.03
		[1.75]	[2.86]
Hospitalisations	0.18	0.14	0.70
		[0.17]	[0.41]

Standard errors in brackets

The results of the analysis of the determinants of latent class membership are shown in Table 7 in Appendix. It is worth noting that having a cognitive impairment is associated with a higher probability of being a frequent user of doctor and GP visits. In the same direction, sensory impaired individuals are associated with a higher probability of visiting a doctor frequently.

## Discussion

This paper analyses the utilisation of health care services by older Europeans with cognitive and sensory impairments. This is carried out by using two different applications of the correlated random effects (CRE) method to describe the variation in the number of visits to any doctor, to the GP, and to the hospital amongst respondents of the SHARE survey. These econometric techniques test the hypothesis that individuals with cognitive and sensory impairments with no regular physical activity will tend to use more health care than people with only one or with none of these impairments in order to partly compensate for the higher depreciation of their health stock. This analysis provides evidence suggesting that, in some cases, cognitive and sensory impairments are associated with higher utilisation of health care services, even after conditioning for other major health conditions. Specifically, the correlated random effects negative binomial model (CCRENB) shows that cognitive impairment is associated with more hospitalisations. Likewise, the latent class approach (LCNB_CRE) finds that amongst high users of health care those with cognitive impairment are significantly associated with more hospitalisations and more GP visits. Additionally, estimates from the LCNB_CRE model indicate a strong, positive and significant association between sensory impairment and the number of hospitalisations amongst low users. Furthermore, the unrestricted models suggest a positive association between suffering both impairments at the same time and the number of doctor and GP visits. Although in some specifications the estimates of the interaction term were statistically significant, the models from which they were obtained are outperformed by the restricted models that omit the Sense-Cog interaction.

On the other hand, the CCRENB shows that individuals with cognitive but no sensory impairment have a negative association with doctor visits. The LCNB_CRE also finds a negative association between cognitive impairment and this type of care amongst low users. These results are not necessarily contrary to the hypothesis tested here as they may be reflecting barriers to outpatient health care access amongst cognitively impaired individuals. Walsh and colleagues suggested that individuals with cognitive impairment may not recognise or be able to communicate health problems requiring outpatient care [41]. One may argue that this lack of outpatient care could lead to more severe illnesses requiring hospitalisations; thus, suggesting that the observed negative association with doctor visits and the positive association with hospitalisations amongst high users of health care could be potentially related. However, testing the existence of substitution and/or complementary behaviours in the use of the different types of health care requires the estimation of a structural demand model which is beyond the scope of this paper.

Regarding the rest of the factors that condition for health need, the results of both econometric models are as predicted by the Grossman theory. Individuals in major health need (higher number of chronic diseases and of daily life limitations) are associated with higher utilisation of health care while individuals with alternative ways of health investment, i.e. regular physical activity, tend to use fewer health care services. Recent studies analysing health care utilisation amongst older Europeans using either SHARE or other surveys have also found these associations [42–45].

In a cross-sectional study using SHARE, Solé-Auro et al. found in their pooled (11 country sample) model a positive association between being an immigrant and visiting the doctor, the GP and the hospital [45]. Interestingly, while the CCRENB model in the present analysis also finds a positive association for the first two types of care, the results of the LCNB_CRE model show that the direction of the association for hospital visits depends on its intensity: the association is positive for low users, but negative for high users.

This paper is subject to three main limitations. First, as with any other study using panel surveys, the results presented here are subject to non-response and attrition bias. However, a recent study using SHARE rejected the hypothesis of significant correlation patterns of missing values and health care utilisation variables [43]. This, nevertheless, does not necessarily apply to the results of the LCNB_CRE model as it was estimated using the balanced panel. Long-term survivors who remain in a panel are likely to be healthier on average than the original sample at the beginning of the panel; this is the source of potential bias in the estimated associations [38]. Second, health care use based on recall of respondents’ past utilisation in the last year may be subject to be underreported. Recall bias may be a particularly important issue amongst cognitively impaired individuals [41]. Unfortunately, without an effective linkage between population surveys and administrative records similar studies will continue to face the same limitation. Third, the models estimated here are not demand functions and treating the three types of health care as independent is a strong assumption. However, the methodology followed in this paper allows the identification of associations between suffering sensory and/or cognitive impairments and using these types of care over and above other individual characteristics. This initial analysis can be complemented with the further estimation of demand functions using structural models that apart from identifying factors associated with the use of health care could also identify price elasticities as well as substitution and complimentary behaviours amongst the types of care studied here. This is, however, beyond the scope of this paper.

## Conclusions

While some evidence suggests that individuals with both sensory and cognitive impairments tend to have more doctor and GP visits that individuals with only one and with none of these impairments, the models that better fit the data did not include the interaction of these impairments. Nevertheless, this analysis shows the existence of a positive association between health care utilisation and cognitive and sensory impairments over and above the existing association of health care utilisation with other major chronic conditions. Given that 24% of the individuals in the sample studied were classified as having both sensory and cognitive impairments, a potential improvement in the efficiency of health care delivery may come from taking a systematic integrated care approach in the treatment of these conditions.

## Appendix

**Table 5:** IRR of time average of covariates in CCRENB

Age	Doctor Visits		GP visits		Hospitalisations	
	1.016*	1.017*	1.029***	1.029***	0.981	0.982
	[0.009]	[0.009]	[0.011]	[0.011]	[0.030]	[0.030]
Married	1.002	1.003	1.003	1.004	0.873	0.873
	[0.025]	[0.025]	[0.035]	[0.035]	[0.077]	[0.077]
Employed	0.945***	0.946***	0.903***	0.904***	0.817***	0.820***
	[0.019]	[0.019]	[0.023]	[0.023]	[0.054]	[0.054]
ln(Income_ppp)	1.005	1.005	0.982***	0.982***	1.000	1.000
	[0.005]	[0.005]	[0.006]	[0.006]	[0.015]	[0.015]
Present smoker	1.011	1.011	0.977	0.977	1.744***	1.744***
	[0.020]	[0.020]	[0.025]	[0.025]	[0.112]	[0.112]
Regular alcohol consumption	0.992	0.992	0.975	0.975	1.076	1.075
	[0.014]	[0.014]	[0.017]	[0.017]	[0.048]	[0.048]
Regular physical activity	0.919***	0.919***	0.896***	0.896***	0.963	0.963
	[0.013]	[0.013]	[0.016]	[0.016]	[0.042]	[0.042]
ADL scale	1.000	1.001	1.019**	1.021**	1.050**	1.053**
	[0.008]	[0.008]	[0.010]	[0.010]	[0.023]	[0.023]
No. of chronic diseases	1.210***	1.210***	1.219***	1.219***	1.181***	1.182***
	[0.006]	[0.006]	[0.007]	[0.007]	[0.017]	[0.017]
Cognitive impairment	1.020	1.055***	1.117***	1.163***	0.974	1.035
	[0.016]	[0.021]	[0.021]	[0.028]	[0.045]	[0.062]
Sensory impairment	1.015	1.038**	1.007	1.039*	1.048	1.091*
	[0.013]	[0.016]	[0.016]	[0.021]	[0.041]	[0.052]
Sense_Cog impairment		0.927***		0.913***		0.879
		[0.026]		[0.031]		[0.072]
SHARE Wave 2	0.976	0.975	0.947	0.946	1.012	1.011
	[0.049]	[0.049]	[0.057]	[0.057]	[0.150]	[0.150]
SHARE Wave 4	0.826***	0.824***	0.733***	0.731***	1.023	1.019
	[0.054]	[0.054]	[0.057]	[0.057]	[0.227]	[0.226]
SHARE Wave 5	0.749***	0.747***	0.703***	0.701***	0.812	0.81
	[0.060]	[0.060]	[0.068]	[0.068]	[0.225]	[0.224]

*IRR* Variables are individual averages over the observed panel. Estimates are reported as incidence-rate ratios. * *p* < 0.10, ** *p* < 0.05, *** *p* < 0.01. Standard errors in brackets

**Table 6:** Health Care utilisation in SHARE: LCNB_CRE with Sense-Cog impairment

	Doctor Visits		GP visits		Hospitalisations	
	[1]	[2]	[3]	[4]	[5]	[6]
*Predisposing factors*	*Low users*	*High users*	*Low users*	*High users*	*Low users*	*High users*
Age	1.012	1.021	1.015	0.99	1.056	0.998
	[0.013]	[0.014]	[0.015]	[0.013]	[0.048]	[0.056]
Age squared	1.000	1.000	1.000	1.000	1.000	1.000
	[0.000]	[0.000]	[0.000]	[0.000]	[0.000]	[0.000]
Male	0.909***	0.991	0.967*	1.013	1.240***	1.182**
	[0.016]	[0.019]	[0.019]	[0.020]	[0.070]	[0.089]
Married	1.057**	1.000	1.032	0.984	1.048	1.157
	[0.025]	[0.023]	[0.027]	[0.023]	[0.081]	[0.132]
Immigrant	1.041	0.978	0.976	0.995	1.213*	0.737*
	[0.037]	[0.038]	[0.035]	[0.037]	[0.123]	[0.124]
Secondary education	1.029	1.011	0.988	0.917***	1.051	1.061
	[0.023]	[0.023]	[0.025]	[0.021]	[0.073]	[0.096]
Tertiary education	1.077***	1.083**	0.920***	0.855***	0.964	1.153
	[0.029]	[0.034]	[0.028]	[0.028]	[0.082]	[0.137]
*Enabling factors*						
Employed	0.923***	0.859***	0.952*	0.846***	0.874	0.816
	[0.024]	[0.025]	[0.026]	[0.027]	[0.103]	[0.104]
ln(income_ppp)	1.025***	0.996	1.015*	0.994	1.001	0.984
	[0.008]	[0.006]	[0.008]	[0.006]	[0.024]	[0.025]
*Health behaviours*						
Present smoker	0.832***	0.936**	0.836***	0.966	0.912	0.748***
	[0.023]	[0.024]	[0.024]	[0.026]	[0.098]	[0.083]
Regular alcohol consumption	0.964**	0.876***	0.961**	0.915***	0.858**	0.837**
	[0.017]	[0.017]	[0.019]	[0.019]	[0.054]	[0.074]
Regular physical activity	0.883***	0.919***	0.907***	0.908***	0.843***	0.761***
	[0.015]	[0.018]	[0.017]	[0.019]	[0.054]	[0.065]
*Need factors*						
ADL scale	1.042**	1.128***	1.011	1.099***	1.271***	1.120**
	[0.017]	[0.015]	[0.023]	[0.012]	[0.040]	[0.060]
Number of chronic diseases	1.338***	1.143***	1.339***	1.114***	1.329***	1.088***
	[0.010]	[0.008]	[0.012]	[0.008]	[0.028]	[0.034]
Cognitive impairment	0.946**	0.975	0.986	1.018	0.865	1.061
	[0.025]	[0.026]	[0.029]	[0.027]	[0.078]	[0.118]
Sensory impairment	0.992	0.960*	0.992	0.973	1.151**	0.885
	[0.019]	[0.021]	[0.021]	[0.023]	[0.075]	[0.081]
Sense_Cog impairment	0.988	1.098***	0.979	1.096***	1.061	1.214
	[0.038]	[0.039]	[0.044]	[0.039]	[0.130]	[0.183]
*Country dummies*						
Austria	1.348***	1.229***	1.114**	1.342***	2.063***	1.074
	[0.069]	[0.060]	[0.055]	[0.068]	[0.259]	[0.197]
Germany	1.982***	1.660***	1.345***	1.502***	1.916***	0.912
	[0.071]	[0.080]	[0.054]	[0.074]	[0.233]	[0.160]
Netherlands	1.132***	1.139***	0.948	0.868***	0.893	0.781
	[0.040]	[0.051]	[0.040]	[0.042]	[0.114]	[0.122]
Spain	1.417***	1.164***	1.181***	1.341***	0.699***	0.619***
	[0.057]	[0.053]	[0.058]	[0.064]	[0.097]	[0.103]
Italy	1.528***	1.495***	1.153***	1.855***	0.832	0.625***
	[0.060]	[0.066]	[0.050]	[0.086]	[0.108]	[0.099]
France	1.673***	1.274***	1.608***	1.335***	1.270**	0.953
	[0.057]	[0.057]	[0.060]	[0.062]	[0.150]	[0.143]
Switzerland	1.124***	1.154**	0.920*	1.102	1.451***	1.395
	[0.049]	[0.068]	[0.045]	[0.070]	[0.197]	[0.290]
Belgium	1.648***	1.434***	1.502***	1.578***	1.286**	0.738**
	[0.052]	[0.058]	[0.053]	[0.068]	[0.140]	[0.107]
N	28,328		26,571		28,736	
ll	−79,599.46		−65,222.37		−14,501.18	
AIC	159,332.92		130,578.75		29,136.36	
BIC	159,792.90		131,053.71		29,597.29	

*IRR* Incidence-rate ratios. * *p* < 0.10, ** *p* < 0.05, *** *p* < 0.01. IRR of time-averaged covariates are not shown. Standard errors in brackets

**Table 7:** Estimated class membership probability (π)

	Doctor visits	GP visits	Hospitalisations
π=	*high users*	*low users*	*low users*
Age	−0.039***	0.001	0.065***
	[0.006]	[0.006]	[0.017]
Married	−0.104	−0.047	0.229
	[0.103]	[0.099]	[0.252]
Employed	−0.272**	0.083	0.389
	[0.133]	[0.128]	[0.297]
ln(Income_ppp)	−0.105**	0.150***	−0.015
	[0.043]	[0.038]	[0.081]
Present smoker	0.203*	−0.203*	−0.773***
	[0.118]	[0.113]	[0.275]
Regular alcohol consumption	0.042	0.134	−0.131
	[0.095]	[0.091]	[0.213]
Regular physical activity	−0.205*	0.265**	−0.037
	[0.113]	[0.106]	[0.244]
ADL scale	0.273**	−0.263***	−0.188
	[0.110]	[0.101]	[0.182]
No. of chronic diseases	0.569***	−0.651***	−0.509***
	[0.044]	[0.045]	[0.078]
Cognitive impairment	0.664***	−0.851***	−0.356
	[0.120]	[0.108]	[0.241]
Sensory impairment	0.330***	−0.151	−0.224
	[0.107]	[0.100]	[0.225]
Constant	2.313***	−0.128	−1.54
	[0.598]	[0.544]	[1.423]

Variables are individual averages over the observed panel. **p* < 0.10, ***p* < 0.05, ****p* < 0.01

## Abbreviations

ADL: Activity Daily Living
CCRE: Conditionally Correlated Random Effects
CCRENB: Conditionally Correlated Random Effects Negative Binomial
CRE: Correlated Random Effects
FE: Fixed effects
GP: General Practitioner
IRRs: Incidence-rate ratios
LCNB_CRE: Latent Class Negative Binomial with correlated random effects
RE: Random Effects
SHARE: Survey of Health, Ageing and Retirement in Europe

## Footnotes

1 We thank an anonymous reviewer for stressing the importance of emphasising the role of regular physical activity in this hypothesis.

2 Due to a relatively high rate of non-response, the imputed values provided in SHARE were used. For simplicity, the average of the imputations provided was used. This simplification does not affect the main results of this paper.
